# Serum Lipid Levels and Dyslipidaemia Prevalence among 2–10 Year-Old Northern Mexican Children

**DOI:** 10.1371/journal.pone.0119877

**Published:** 2015-03-20

**Authors:** Maria del Mar Bibiloni, Rogelio Salas, Hilda I. Novelo, Jesús Z. Villarreal, Antoni Sureda, Josep A. Tur

**Affiliations:** 1 Research Group on Community Nutrition and Oxidative Stress, University of Balearic Islands and CIBEROBN (Physiopathology of Obesity and Nutrition), Palma de Mallorca, Spain; 2 Faculty of Public Health Nutrition, Autonomous University of Nuevo León, Monterrey, Mexico; 3 Department of Health of the State of Nuevo León, Monterrey, Mexico; Innsbruck Medical University, AUSTRIA

## Abstract

**Background and Aims:**

The increase in overweight and obese children may be linked to increased rates of dyslipidaemia. The aim was to assess the prevalence of dyslipidaemia and associated risk factors among the Northern Mexican child population.

**Methods and Results:**

Four hundred and fifty-one subjects aged between 2 and 10 (47.5% girls) took part in the Nuevo León State Survey of Nutrition and Health 2011–2012. According to the 2011 Expert Panel on Integrated Guidelines for Cardiovascular Health and Risk Reduction in Children and Adolescents, serum lipid levels (mg/dL) were categorized into three subgroups (acceptable, borderline-high/low or high/low) as follows: TChol: acceptable <170, borderline-high 170–199, high ≥200; LDL-chol: acceptable <110, borderline-high 110–129, high ≥130; non-HDL-chol: acceptable <120, borderline-high 120–144, high ≥145; HDL-chol: acceptable >45, borderline-low 40–45, low <40; and TG: acceptable <75, borderline-high 75–99, high ≥100 in ≤9 year-old children, and acceptable <90, borderline-high 90–129, and high ≥130 in 10 year-old children. The overall prevalence of borderline-high + high TG, non-HDL-chol, TChol, and LDL-chol was 63.0%, 44.1%, 43.5%, and 29.9%, respectively. The overall prevalence of borderline-low + low HDL-chol was 46.3%. The overall frequency of dyslipidaemia was 54.3%. Thirteen children (2.9%) had all five symptoms of dyslipidaemia. The most common dyslipidaemia was high TG in combination (26.2%) and in isolation (10.6%).

**Conclusions:**

Half of the children had at least one abnormal lipid concentration. A high TG level was the most frequent dyslipidaemia. Obesity was associated with the occurrence of at least one abnormal lipid level. These findings emphasize the need to pay further attention to the prevention of cardiovascular disease and obesity from an early age.

## Introduction

Dyslipidaemia is abnormal amounts of lipid (i.e. cholesterol and fatty acids) and/or lipoprotein in the blood. Dyslipidaemia may be related to other diseases (secondary dyslipidaemia) or to the interaction between genetic predisposition and environmental factors, such as unhealthy diet, lack of physical activity and/or weight gain [1], with obesity being the most common [2]. The prevalence of overweight and obesity in Mexican pre-school children (<5 years old) and school children (between 5–11 years old) in 2012 was 9.7% and 34.4%, respectively [3]. Child overweight and obesity rates in Mexico are among the highest in the Organisation for Economic Co-operation and Development (OECD) area [4]. Moreover, Mexican-Americans (born and raised in the United States of America and Mexican immigrants (born in Mexico and raised in the United States of America) are more likely to be obese than their Mexican peers (born and raised in Mexico) [5]. The increase in overweight and obesity in children may be linked to increased rates of dyslipidaemia [2].

Several guidelines have recommended screening children over 2 years old if they have cardiovascular risk factors, a family history of premature cardiovascular disease (CVD) or dyslipidaemia, are overweight or obese, have other indications of insulin resistance syndrome, or have no available family history [6–8]. Recently, the 2011 Expert Panel on Integrated Guidelines for Cardiovascular Risk Reduction on lipid screening in Childhood and Adolescence [9] endorsed universal screening of all children between 9 and 11 years old, a stable time for lipid assessment in children as it precedes the onset of puberty for most of them, to identify children with dyslipidaemia at an early age. There is no evidence that diagnosis and treatment in childhood and adolescence improves long-term primary outcomes; however, using family history of premature CVD or cholesterol disorders as the primary factor in determining lipid screening for children misses out approximately 30–60% of the children with dyslipidaemia [9]. Moreover, previous studies showed that in ~50% of children with high lipid and lipoprotein levels, abnormal levels will persist over time [10–13]. Adverse serum lipid levels in children predict dyslipidaemia in adulthood and adverse levels of non-high-density lipoprotein cholesterol (non-HDL-chol) are also related to non-lipid cardiovascular risk factors in adulthood [14]. So, recent epidemic of obesity and the metabolic syndrome in Mexican youth highlight the need to identify children at enhanced risk for atherosclerosis. Therefore, the aim of this study was to assess the prevalence of dyslipidaemia and associated risk factors among children (between 2–10 years old) living in Northern Mexico.

## Methods

### Study design

The study was a population-based cross-sectional nutritional survey carried out in the State of Nuevo León, Mexico (2011–2012).

### Study population, recruitment and approval

This study is part of the State Survey of Nutrition and Health—Nuevo León 2011/2012 (EESN-NL 2011/2012). The EESN-NL 2011/2012 was designed by the National Institute of Statistics and Geography (INEGI) to obtain information about the health and nutritional status of the population living in the State of Nuevo León. The state was divided into four regions: northern, central, southern and the metropolitan area. Neighbourhood blocks were randomly selected and all subjects in all households were invited to take part in the survey (n = 1180 per region; total = 4720 households invited). A target of 1059 households per region was assessed (89.7% participation) using the household as the sampling unit and an average of 2 interviews per household. The sample size was considered to be large enough to detect risk factors at regional level that had, at least, a prevalence of 8%, with a relative error calculation of 15% and a non-response rate of 40%. This sample size also calculated a prevalence of 9.0% in individuals aged between 0 and 9 years old, 10.0% in those aged from 10 to 19 years old, 5.0% in 20 to 59 year-olds, and 17% of the over 60 year old population. Information was obtained from 4,236 households and 7,290 individuals (1,372 0–9 year-olds; 1,319 10–19 year-olds; 3,125 20–59 year-olds; 1,474 ≥60 year-olds). One participant in the same age group living in the same household was selected. There were no socioeconomic differences between people that participate in the survey and those that declined. Participation was similar in all age groups.

### Sample selection

**Fig 1:**
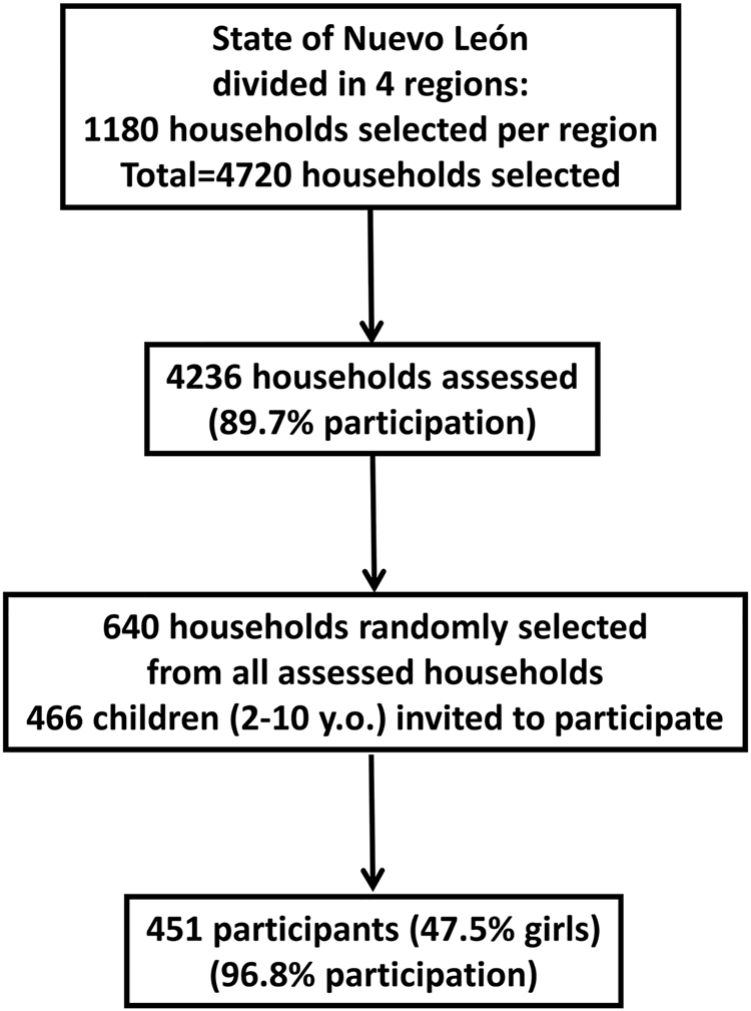
Flow-chart for population recruitment.

A sample size of 640 households randomly selected from all assessed households was considered sufficient to detect risk factors with 95% confidence and a precision rate of 10%, and 466 children aged between 2 and 10 years were invited to participate. The analysis was limited to children who provided a fasting blood test with no missing data (*n* = 451, 47.5% girls, 96.8% participation) (Fig. 1).

### Ethics

The study was conducted according to the guidelines laid down in the Declaration of Helsinki, and all procedures involving human subjects were approved by the Scientific Technical Committee and the Ethics Committee of the Public Health and Nutrition Faculty at the Autonomous University of Nuevo León before the study began. Informed written consent was obtained from the next of kin, carers, or guardians of the adults and minors involved in the study.

### Anthropometric measurements

Height was determined to the nearest millimetre using a mobile stadiometer (SECA 213, Birmingham, United Kingdom), with the subject’s head in the Frankfurt plane. Body weight was determined to the nearest 100 g using a digital scale (Seca 813, Hamburg, Germany). Height and weight measures were used to calculate body mass index (BMI, kg/m^2^). Weight and/or height were missing for thirty four subjects.

### Nutritional status definition

Underweight, normal-weight, overweight and obesity were determined based on gender- and age-specific BMI cut-offs developed and proposed for international comparisons by Cole et al. [15,16], and also recommended by the International Obesity Task Force (IOTF).

### Biochemical measurements

Venous blood samples were obtained from the antecubital vein in suitable vacutainers after 12 hour overnight fasting. Blood samples were centrifuged at 900 g at 4°C for 10 minutes. Serum total cholesterol (TChol), high-density lipoprotein cholesterol (HDL-chol) and triglycerides (TG) were determined by enzymatic methods according to manufacturer’s recommendation (Roche Diagnostics, Mexico D.F.) using the Cobas 6000 analyser series (F. Hoffmann-La Roche Ltd, Basel, Switzerland) by the “Dr. Bernardo Sepúlveda” laboratory at the Metropolitan Hospital under the Ministry of Health of Nuevo León, Mexico. TChol was measured by means of an enzymatic colorimetric method. Cholesterol esters were cleaved by the action of cholesterol esterase to yield free cholesterol and fatty acids. Cholesterol oxidase then catalyzes the oxidation of cholesterol to cholest-4-en-3-one and hydrogen peroxide. In the presence of peroxidase, the hydrogen peroxide formed effects the oxidative coupling of phenol and 4-aminophenazone to form a red quinoneimine dye. The color intensity of the dye formed was directly proportional to the cholesterol concentration. It was determined by measuring the increase in absorbance. TG in the sample were hydrolyzed by lipoprotein lipase to glycerol and fatty acids. The glycerol was phosphorylated to glycerol-3-phosphate by glycerol kinase and subsequently catalyzed by glycerol oxidase to form dihydroxyacetone phosphate and hydrogen peroxide. The hydrogen peroxide was then reacted in the presence of peroxidase to form a quinoneimine dye and the absorbance measured at 500 nm. Serum samples were hydrolysed with cholesterol esterase to obtain total cholesterol. 4-aminoantipyrine and cholesterol oxidase reacts with cholesterol to form quinoneimine. The absorbance of quinoneimine was determined at 500 nm. HDL-chol was measured serum using the cholesterol oxidase cholesterol method (Roche Diagnostics, Mexico D.F.) after precipitation of non-HDL-chol with magnesium/dextran. LDL-chol was calculated from measurements of TChol, total triglycerides, and HDL-chol by the Friedewald equation modified by DeLong et al [17]: LDL-chol = TChol—HDL-chol—TG/5. Calibrations were performed with commercial calibrators and according to manufacturer’s instructions [18], and the coefficients of variations (CV) were 1.1 (TChol), 0.4 (HDL-chol), and 0.9 (TG) for Precinorm; 0.9 (TChol), 1.0 (HDL-chol), and 0.8 (TG) for Precipath; 1.1 (TChol), 0.6 (HDL-chol), and 1.1 (TG) for human serum 1; and 0.7 (TChol), 0.7 (HDL-chol), and 0.7 (TG) for human serum 2. Accuracy and precision were under the external surveillance of the Programa de Aseguramiento de la Calidad (PACAL, Mexico), with ISO 9001:2008 and ISO/IEC 17043/2010 certification, which grants scores of variance index (PIV) of 0 (TChol), 37 (HDL-chol), and 6 (TG).

### Children’s’ definitions for adverse serum lipid levels and dyslipidaemia

According to the 2011 Expert Panel on Integrated Guidelines for Cardiovascular Health and Risk Reduction in Children and Adolescents [9], serum lipid levels (mg/dL) were categorized into three subgroups (acceptable, borderline-high/low or high/low) as follows: TChol: acceptable <170, borderline-high 170–199, high ≥200; LDL-chol: acceptable <110, borderline-high 110–129, high ≥130; non-HDL-chol: acceptable <120, borderline-high 120–144, high ≥145; HDL-chol: acceptable >45, borderline-low 40–45, low <40; and TG: acceptable <75, borderline-high 75–99, high ≥100 in ≤9 year-old children, and acceptable <90, borderline-high 90–129, and high ≥130 in 10 year-old children.

Dyslipidaemia was defined as the presence of one or more of the following conditions: TChol ≥200 mg/dL, LDL-chol ≥130 mg/dL, non-HDL-chol ≥145 mg/dL, HDL-chol <40 mg/dL, and TG ≥100 mg/dL in ≤9 year-old children and TG ≥130 mg/dL in 10 year-old children.

### Selection of mother and child pairs

Two hundred and thirty-four one-to-one mother and child pairs (43.3%) were indentified in the study. Children with more than one possible mother in a household were excluded from the one-to-one matches.

### Maternal dyslipidaemia definition

Maternal dyslipidaemia was defined as the presence of one or more of the following conditions (mg/dL): TChol ≥240, LDL-chol ≥160, HDL-chol <40, or TG ≥200 [19].

### Statistical analysis

Analyses were performed with the SPSS statistical software package version 21.0 (SPSS Inc., Chicago, IL, USA). The level of significance for acceptance was *P* <0.05. Significant differences in prevalence were calculated by means of χ^2^ test or the two-tailed Fisher’s exact test where the expected frequency in any cell was less than 5. Differences between group means were tested by the unpaired Students’ *t-*test, and by analysis of variance. Multivariate analyses (multiple logistic regressions considering the simultaneous effect of each explanatory variable adjusted for gender and age) were used to assess the association between gender, age, BMI status and maternal dyslipidaemia (independent variables) and children’s dyslipidaemia (dependent variable).

## Results

Table 1 shows the serum lipid and lipoprotein levels of the participants. There were significant differences in HDL-chol and TG between boys and girls, but no differences in TChol, LDL-chol and non-HDL-chol between genders were found. Girls showed lower HDL-chol levels than boys aged between 2 and 4 years old, and higher TG levels than boys aged between 5 and 7 years old. 2–4 year-old girls also showed lower HDL-chol levels than those between 5 and 7 years old. Boys between 8 and 10 years old showed higher TG levels than 2–7 year-olds.

**Table 1:** Serum lipids concentrations (mg/dL) among Northern Mexican children^1^,^2^.

	Age group (years)				
	All	2–4	5–7	8–10	*P*†
*n (boys/girls)*	237/214	53/48	69/78	115/88	
TChol					
All	167.4 ± 33.3	164.8 ± 31.1	165.8 ± 32.7	169.8 ± 34.7	0.377
	(164.3–170.5)	(158.7–171.0)	(160.5–171.2)	(164.3–170.5)	
Boys	166.5 ± 33.0	161.4 ± 27.8	163.1 ± 30.4	170.9 ± 36.2	0.131
	(162.3–170.7)	(153.7–169.1)	(155.8–170.4)	(164.2–177.6)	
Girls	168.3 ± 33.6	168.6 ± 34.2	168.2 ± 34.7	168.3 ± 32.7	0.998
	(163.8–172.9)	(158.7–178.6)	(160.4–176.1)	(161.3–175.2)	
*P*‡	0.558	0.245	0.340	0.592	
LDL-chol					
All	98.0 ± 26.9	100.6 ± 29.0	96.7 ± 25.3	97.6 ± 27.1	0.506
	(95.5–100.5)	(94.9–106.4)	(92.6–100.8)	(93.8–101.3)	
Boys	96.3 ± 26.6	98.5 ± 29.6	93.7 ± 21.3	96.9 ± 28.0	0.578
	(92.9–99.7)	(90.4–106.7)	(88.6–98.8)	(91.7–102.0)	
Girls	99.8 ± 27.3	102.9 ± 28.4	99.3 ± 28.3	98.5 ± 26.0	0.650
	(96.1–103.5)	(94.7–111.2)	(93.0–105.7)	(93.0–104.0)	
*P*‡	0.172	0.448	0.169	0.680	
HDL-chol					
All	46.9 ± 11.7	45.1 ± 12.0	48.5 ± 10.3	46.7 ± 12.3	0.065
	(45.8–48.0)	(42.7–47.4)	(46.8–50.2)	(45.0–48.4)	
Boys	48.0 ± 11.8	47.4 ± 9.7	48.8 ± 10.1	46.7 ± 13.5	0.755
	(46.4–49.5)	(44.7–50.1)	(46.4–51.3)	(45.2–50.2)	
Girls	45.8 ± 11.5	42.5 ± 13.7	48.3 ± 10.6	45.4 ± 10.5	0.020
	(44.2–47.3)	(38.5–46.5)	(45.9–50.6)	(43.2–47.6)	
*P*‡	0.049	0.038	0.739	0.176	
Non-HDL-chol					
All	120.5 ± 32.8	119.8 ± 31.5	117.3 ± 31.5	123.1 ± 34.3	0.260
	(117.4–123.5)	(113.6–126.0)	(112.2–122.4)	(118.3–127.8)	
Boys	118.6 ± 32.4	114.0 ± 28.3	114.3 ± 28.9	123.2 ± 35.6	0.097
	(114.4–122.7)	(106.2–121.8)	(107.3–121.2)	(116.7–129.8)	
Girls	122.6 ± 33.3	126.2 ± 33.8	120.0 ± 33.6	122.9 ± 32.9	0.599
	(118.1–127.0)	(116.3–136.0)	(112.4–127.6)	(115.9–129.8)	
*P*‡	0.197	0.052	0.272	0.941	
TG					
All	104.1 ± 55.0	96.9 ± 46.9	94.4 ± 46.8	114.8 ± 62.1	0.001
	(87.6–106.1)	(87.6–106.1)	(86.8–102.1)	(106.2–123.3)	
Boys	98.5 ± 51.7	88.3 ± 31.9	85.4 ± 35.4	111.0 ± 63.3	0.001
	(91.8–105.1)	(79.5–97.1)	(76.8–93.9)	(99.3–122.7)	
Girls	110.4 ± 57.9	106.4 ± 58.1	102.5 ± 53.9	119.7 ± 60.4	0.138
	(102.6–118.2)	(89.5–123.2)	(90.3–114.6)	(106.9–132.5)	
*P*‡	0.021	0.060	0.023	0.324	

Abbreviations: TChol, total cholesterol; LDL-chol, low-density lipoprotein cholesterol; HDL-chol, high-density lipoprotein cholesterol; non-HDL-chol, non-high-density lipoprotein cholesterol; TG, tryglicerides.

^1^Values are mean ± standard deviation (95% confidence interval).

^2^Significant differences between age groups by †ANOVA. Significant differences between boys and girls by ‡unpaired Student *t* test.

Table 2 shows the prevalence of risk categories for lipid serum and lipoprotein levels among children. The overall prevalence of borderline-high + high TG, non-HDL-chol, TChol and LDL-chol was 63.0%, 44.1%, 43.5% and 29.9%, respectively. TG prevalence was higher among girls (68.2%) than boys (58.2%). The overall frequency of borderline-low + low HDL-chol was 46.3%. This frequency was lower in children aged between 5 and 7 years old (37.4%) than in those aged between 2 and 4 (52.5%) and those aged between 8 and 10 (49.8%).

**Table 2:** Prevalence of risk categories for plasma lipid and lipoprotein concentrations (mg/dL) among Northern Mexican children.

	Total					Boys					Girls				
	All	2–4	5–7	8–10	*P*	All	2–4	5–7	8–10	*P*	All	2–4	5–7	8–10	*P*
*n*	451	101	147	203		237	53	69	115		214	48	78	88	
TChol (mg/dL), %															
Acceptable (<170)	56.5	58.4	57.8	54.7	0.876	56.5	62.3	59.4	52.2	0.530	56.5	54.2	56.4	58.0	0.994
Borderline-high (170–199)	26.8	26.7	27.2	26.6		28.3	26.4	29.0	28.7		25.2	27.1	25.6	23.9	
High (≥200)	16.6	14.9	15.0	18.7		15.2	11.3	11.6	19.1		18.2	18.8	17.9	18.2	
Borderline-high + High (≥170)	43.5	41.6	42.2	45.3	0.768	43.5	37.7	40.6	47.8	0.400	43.5	45.8	43.6	42.0	0.913
LDL-chol (mg/dL), %															
Acceptable (<110)	70.1	69.3	69.4	70.9	0.569	70.9	69.8	73.9	69.6	0.600	69.2	68.8	65.4	72.7	0.697
Borderline-high (110–129)	17.7	15.8	21.1	16.3		18.1	17.0	20.3	17.4		17.3	14.6	21.8	14.8	
High (≥130)	12.2	14.9	9.5	12.8		11.0	13.2	5.8	13.0		13.6	16.7	12.8	12.5	
Borderline-high + High (≥110)	29.9	30.7	30.6	29.1	0.936	29.1	30.2	26.1	30.4	0.805	30.8	31.3	34.6	27.3	0.592
HDL-chol (mg/dL), %															
Acceptable (>45)	53.7	47.5	62.6	50.2	0.083	54.9	52.8	63.8	50.4	0.192	52.3	41.7	61.5	50.0	0.132
Borderline-low (40–45)	19.5	22.8	17.7	19.2		21.1	28.3	17.4	20.0		17.8	16.7	17.9	18.2	
Low (<40)	26.8	29.7	19.7	30.5		24.1	18.9	18.8	29.6		29.9	41.7	20.5	31.8	
Borderline-low + Low (≤45)	46.3	52.5	37.4	49.8	0.027	45.1	47.2	36.2	49.6	0.201	47.7	58.3	38.5	50.0	0.081
Non-HDL-chol (mg/dL), %															
Acceptable (<120)	55.9	56.4	59.2	53.2	0.789	58.6	62.3	60.9	55.7	0.220	52.8	50.0	57.7	50.0	0.559
Borderline-high (120–144)	21.5	21.8	21.1	21.7		19.8	22.6	23.2	16.5		23.4	20.8	19.2	28.4	
High (≥145)	22.6	21.8	19.7	25.1		21.5	15.1	15.9	27.8		23.8	29.2	23.1	21.6	
Borderline-high + High (≥120)	44.1	43.6	40.8	46.8	0.534	41.4	37.7	39.1	44.3	0.653	47.2	50.0	42.3	50.0	0.555
TG (mg/dL), %															
Acceptable (<75/90)	37.0	36.6	41.5	34.0	0.463	41.8	41.5	46.4	39.1	0.676	31.8	31.3	37.2	27.3	0.646
Borderline-high (75–99/90–129)	26.2	29.7	25.2	25.1		25.3	28.3	26.1	23.5		27.1	31.3	24.4	27.3	
High (≥100/130)	36.8	33.7	33.3	40.9		32.9	30.2	27.5	37.4		41.1	37.5	38.5	45.5	
Borderline-high + High (≥75/90)†	63.0	63.4	58.5	66.0	0.355	58.2	58.5	53.6	60.9	0.627	68.2	68.8	62.8	72.7	0.391

Abbreviations: TChol, total cholesterol; LDL-chol, low-density lipoprotein cholesterol; HDL-chol, high-density lipoprotein cholesterol; non-HDL-chol, non-high-density lipoprotein cholesterol; TG, tryglicerides. Significant differences between age groups by χ^2^ test.

†Significant differences between boys and girls by χ^2^ test (*P* < 0.05).

Table 3 shows dyslipidaemia components among children (high TChol, LDL-chol, non-HDL-chol, and TG; and low HDL-chol) according to gender and BMI status. The overall prevalence of dyslipidaemia was 54.3%: 21.5% had 1 component, 16.2% had 2 components, and 13.7% had 3–4 components. Thirteen children (2.9%) had all five components of dyslipidaemia. There were no statistically significant differences in the frequency of dyslipidaemia between boys (50.6%) and girls (58.4%). The overall criteria for dyslipidaemia were met by 46.3%, 49.4%, 63.8% and 73.5% of underweight, normal-weight, overweight and obese children, respectively. High TG prevalence was greater among underweight girls (35.0%) than boys (12.5%). Low HDL-chol was also higher among underweight and normal-weight girls than boys. The criteria for high TG, non-HDL-chol, TChol and LDL-chol were met by 48.9%, 27.7%, 19.1% and 8.5% of overweight children and 59.2%, 38.8%, 18.4 and 16.3% of obese children, respectively. The prevalence of low HDL-chol was 36.2% in overweight children and 55.1% in obese children.

**Table 3:** Prevalence of dyslipidaemia components among Northern Mexican children^1^.

	*n* (%)	TChol ≥200mg/dL	LDL-chol ≥130mg/dL	HDL-chol <40mg/dL	non-HDL-chol ≥145mg/dL	TG ≥100/130mg/dL	Dyslipidaemia
*Total*	451 (100.0)	16.6	12.2	26.8	22.6	36.8	54.3
*Gender*							
Boys	237 (52.5)	15.2	11.0	24.1	21.5	32.9	50.6
Girls	214 (47.5)	18.2	13.6	29.9	23.8	41.1	58.4
*BMI status*							
Underweight	80 (19.2)	15.0	8.8	23.8§	16.3†	23.8§	46.3‡
Boys	40 (18.4)	17.5	7.5	12.5* ‡	17.5†	12.5* ‡	35.0* ‡
Girls	40 (20.0)	12.5	10.0	35.0	15.0	35.0†	57.5
Normal-weight	241 (57.8)	14.9	12.0	19.5	19.5	32.8	49.4
Boys	129 (59.4)	12.4	9.3	14.7*	17.8	31.0	44.2
Girls	112 (56.0)	17.9	15.2	25.0	21.4	34.8	55.4
Overweight	47 (11.3)	19.1	8.5	36.2	27.7	48.9	63.8
Boys	24 (11.1)	16.7	8.3	29.2	20.8	37.5	58.3
Girls	23 (11.5)	21.7	8.7	43.5	34.8	60.9	69.6
Obesity	49 (11.8)	18.4	16.3	55.1	38.8	59.2	73.5
Boys	24 (11.1)	16.7	20.8	66.7	45.8	58.3	79.2
Girls	25 (12.5)	20.0	12.0	44.0	32.0	60.0	68.0

Abbreviations: TChol, total cholesterol; LDL-chol, low-density lipoprotein cholesterol; HDL-chol, high-density lipoprotein cholesterol; non-HDL-chol, non-high-density lipoprotein cholesterol; TG, tryglicerides.

^1^Significant differences between boys and girls by χ^2^ test or Fisher’s exact test:

*P<0.05.

Significant differences between BMI status groups by χ^2^ test:

†*P*<0.05

‡*P*<0.01

§ *P*<0.001

Table 4 shows that high TG was the most common component of dyslipidaemia, in isolation (10.6%) or combined (26.2%) with other dyslipidaemia components. Low HDL-chol was the second isolated component of lipid abnormality (10.0%) and the third combined component (16.8%), whereas the combination of high TG and low HDL-chol was observed in 9.5% of the participants (*n* = 43). The frequency of dyslipidaemia was higher among children whose mothers showed high TChol levels (≥240 mg/dL, 69.0%) than those whose mothers were normocholesterolemic (<240 mg/dL, 49.3%; *P* < 0.05) (data not shown). Moreover, high TChol and LDL-chol were also greater amongst children whose mothers were hypercholesterolemic, rather than normocholesterolemic.

**Table 4:** Patterns of isolated and combined dyslipidaemias among Northern Mexican children^1^.

Dyslipidaemias	Isolated^2^	Combined^3^
TChol	3 (0.7)	72 (16.0)
LDL-chol	0 (0.0)	55 (12.2)
HDL-chol	45 (10.0)	76 (16.8)
non-HDL-chol	1 (0.2)	101 (22.4)
TG	48 (10.6)	118 (26.2)
TG + HDL-chol	43 (9.5)	―
TG + non-HDL-chol	11 (2.4)	―
Non-HDL-chol + TChol	10 (2.2)	―
TG + HDL-chol + non-HDL-chol	10 (2.2)	―
TG + TChol + non-HDL-chol	12 (2.7)	―
TChol + LDL-chol + non-HDL-chol	13 (2.9)	―
TG + TChol + LDL-chol + non-HDL-chol	19 (4.2)	―

Abbreviations: TChol, total cholesterol; LDL-chol, low-density lipoprotein cholesterol; HDL-chol, high-density lipoprotein cholesterol; non-HDL-chol, non-high-density lipoprotein cholesterol; TG, tryglicerides.

^1^Values are *n* (%).

^2^Considering one factor.

^3^Considering 2 or more factors.

Logistic regression analysis with age, gender, BMI status and mother’s dyslipidaemia covariables (Table 5) showed that the risk of dyslipidaemia was associated with BMI status after adjustment for gender and age. Obese children were significantly more likely to have at least one abnormal lipid level (odds ratio: 2.79; 95% confidence interval: 1.41–5.55) than their normal-weight counterparts. None of the other covariables considered in this study (i.e. gender, age, and maternal dyslipidaemia) were significantly associated with dyslipidaemia.

**Table 5:** Characteristics of Northern Mexican children with and without diagnosis of dyslipidaemia^1^.

	Without dyslipidaemia		With dyslipidaemia		Gender- and age-adjusted OR (95% CI)†‡§
	*n*	%	*n*	%	
*Age group (years)*†					
2–4	47	46.5	54	53.5	1.00 (ref.)
5–7	74	50.3	73	49.7	0.84 (0.51–1.40)
8–10	85	41.9	118	58.1	1.23 (0.76–1.99)
*Gender*‡					
Boys	117	49.4	120	50.6	1.00 (ref.)
Girls	89	41.6	125	58.4	1.37 (0.95–1.99)
*BMI status*†‡					
Underweight	43	53.8	37	46.3	0.88 (0.53–1.47)
Normal-weight	122	50.6	119	49.4	1.00 (ref.)
Overweight	17	36.2	30	63.8	1.76 (0.92–3.39)
Obesity	13	26.5	36	73.5	2.79 (1.41–5.55)**
*Mother’s dyslipidaemia*†‡§					
TChol					
<240	104	50.7	101	49.3	1.00 (ref.)
≥240	9	31.0	20	69.0	2.02 (0.83–4.96)
LDL-chol					
<160	101	47.9	110	52.1	1.00 (ref.)
≥160	12	52.2	11	47.8	1.01 (0.39–2.63)
HDL-chol					
≥40	71	50.0	71	50.0	1.00 (ref.)
<40	42	45.7	50	54.3	1.24 (0.70–2.19)
TG					
<200	96	50.5	94	49.5	1.00 (ref.)
≥200	17	38.6	27	61.4	1.94 (0.92–4.11)
No. dyslipidaemia components					
<1	59	52.2	54	47.8	1.00 (ref.)
≥1	54	44.6	67	55.4	1.43 (0.82–2.49)

Abbreviations: OR, odds ratio; CI, confidence interval; BMI, body mass index; TChol, total cholesterol; LDL-chol, low-density lipoprotein cholesterol; HDL-chol, high-density lipoprotein cholesterol; TG, tryglicerides.

^1^Logistic regression analysis considering the effect of one explanatory variable after adjustment for childrens’ †gender and ‡age (continuous variable), and §mothers’ age (continuous variable).

## Discussion

The main finding of this study was that 54.3% of children between 2 and 10 years old living in the State of Nuevo León, Mexico have at least one abnormal lipid concentration. In spite of the differences in studied ages and the cut-off values for serum lipid level that hampered direct comparison across studies, this prevalence of dyslipidaemia is higher than in previous reported data for children [20,21]. However, alterations in lipid profile have also been recorded in more than half of some Latin American paediatrics’ patients [22–24]. High TG levels were the most common dyslipidaemia in this study, similar to previous data obtained for both Latin American [20,25] and non-Latin American [26,27] children and adolescents, in spite of the fact that low HDL-chol levels (<35 mg/dL) have also been reported as the most prevalent dyslipidaemias amongst other Mexican child and adolescent populations [28].

Gender differences in lipid concentrations were also observed among Northern Mexican children. Girls showed lower HDL-chol than boys aged between 2 and 4 years old (42.5 mg/dL and 47.4 mg/dL, respectively) and higher TG levels than 5 to 7 year-old boys (102.5 mg/dL and 85.4 mg/dL, respectively). The mean HDL-chol was also lower among girls (51.9 mg/dL) than boys (55.1 mg/dL) in 4–11 year-old US children [29], and the mean TG was also higher among 6–12 year-old Mexican girls (126.4 mg/dL) than boys (111.6 mg/dL) [30]. However, in this study there were no differences in the levels of dyslipidaemia components between boys and girls. The prevalence of high non-HDL-chol was also similar among 6–19 year-old US boys (11.2%) and girls (10.1%) [31]. However, controversial results in gender differences in TChol [25,26,28, 32,33], HDL-chol [25,26,28,32], LDL-chol [25–27,32,33] and TG [25,26,28,30,32] levels have been found in the literature. In this study, the mean TG also increased with age in boys. Differences in prevalence of several abnormal lipid levels among age groups [32,34] or Tanner stages [28] have previously been reported.

Obese children showed the highest prevalence of dyslipidaemia, as well as high TG and non-HDL-chol and low HDL-chol levels. Obesity is associated with increased rates of dyslipidaemia and other cardiovascular risk factors [35]. Higher values of TChol [28,36,37], LDL-chol [28,36,37], non-HDL-chol [31] and TG [28,36–38], and lower levels of HDL-chol [28,36] have been also reported in other overweight and obese paediatric populations rather than in normal-weight subjects.

The 2011 Expert Panel on Integrated Guidelines for Cardiovascular Risk Reduction on lipid screening in Childhood and Adolescence [9] recommended lipid screening in children under 9 years old if they have parents with a total cholesterol of ≥240 mg/dL or known dyslipidaemia. Accordingly, the frequency of dyslipidaemia was higher among children whose mothers were hypercholesterolemic than normocholesterolemic peers, in spite of the fact that this association lost its statistical significance in the multivariate analysis after adjustment for children’s gender and age, and mother’s age.

Dyslipidaemia has traditionally been associated with dietary fat intakes. However, it has recently been associated with the consumption of added sugars in adults [39] and adolescents [40], and also high serum TG [41] and low HDL-chol [42] in children. Among children between 5 and 11 years old, intake of caloric sugary beverages increased considerably over the 1999–2012 period in Mexico [43]. In 2012, 17.5% of the total daily caloric intake came from beverages (caloric fizzy drinks, high-fat milk and flavoured milk beverages) among children and adolescents aged between 1 and 19 years old [43]. Therefore, caloric sugary beverages may be also associated with the high prevalence of dyslipidaemia observed in Mexico’s child population. Moreover, in Brazilian preschoolers who less frequently consumed foods in a ‘mixed diet’ dietary pattern (i.e. all food groups following the principles of a healthy diet) showed higher risk of high concentrations of LDL-chol when compared with those with more frequent consumption of this dietary pattern [44]. So, unhealthy diets with sedentary lifestyle habits, which are below the recommendations among Mexican children [45], may explain the high lipid abnormalities levels among them. Therefore, more early intervention to encourage appropriate nutrition and physical activity at an early age could be relevant strategies to prevent and/or reduce the high risk of atherosclerosis in this population. Inappropriate habits that are incorporated in childhood and that increase in adolescence may augment the appearance of diseases in adulthood.

## Strengths and Limitations

This study has several strengths. Firstly, it is often difficult to obtain a fasting lipid profile in large population surveys [46,47] and even more so in children. Secondly, the large, diverse and complex evidence base that addresses cardiovascular disease risk beginning in childhood, and the absence of decades-long event-driven clinical trials, requires consideration of substantial and consistent evidence from observational studies, developing a chain of evidence [48]. This study also has limitations. ApoB and apoA-1 levels were not measured. However, most, but not all, studies point out that measurement of apoB and apoA-1 for universal screening provides no additional advantages over measuring non-HDL-chol, LDL-chol and HDL-chol [49].

## Conclusions

Despite the early age of the group under study, half of them already showed high prevalence of dyslipidaemia, which is an important risk factor for cardiovascular disease. High TG level was the most common dyslipidaemia. Obesity was associated with the prevalence of at least one abnormal lipid level. This emphasises the need to pay further attention to the prevention of cardiovascular disease and obesity already in children also in the North of Mexico. These findings also provide useful information in planning programs targeting the prevention of CVD from childhood.
